# US FDA’s Dose Optimization Postmarketing Requirements and Commitments of Oncology Approvals and the Impact on Product Labels from 2010 to 2022: An Emerging Landscape from Traditional to Novel Therapies

**DOI:** 10.1007/s43441-023-00606-1

**Published:** 2024-01-05

**Authors:** Joseph M. Gendy, Naomi Nomura, Jeffrey N. Stuart, Gideon Blumenthal

**Affiliations:** Global Regulatory Affairs, Merck & Co., Inc., 126 E. Lincoln Avenue, Rahway, NJ 07065 USA

**Keywords:** Dose optimization, Postmarketing studies, US Food and Drug Administration, Drug development, Oncology, Product labeling

## Abstract

**Background:**

Dose optimization is a focal point of many US Food and Drug Administration (FDA) drug approvals. We sought to understand the impact of the FDA’s Postmarketing Commitments/Postmarketing Requirements (PMCs/PMRs) on dose optimization and prescriber labeling for oncology drugs.

**Methods:**

Publicly available information was aggregated for all FDA oncology drug approvals between January 1, 2010, and December 31, 2022. Study completion dates were compared to product labeling before and after PMC/PMR fulfillment dates to evaluate labeling changes associated with dose-related PMCs/PMRs. Data were analyzed individually (2010–2015 and 2016–2022) due to differences in available information.

**Results:**

From 2010 to 2015, 14 of 42 (33.3%) new molecular entities (NMEs) had dose-related PMCs/PMRs, with 6 of 14 (42.9%) resulting in a relevant label change. From 2016 to 2022, of the 314 new or supplemental applications approved, 21 had dose-related PMCs/PMRs (6.7%), which trended upward over time; 71.4% of dose-related PMCs/PMRs were NMEs. Kinase inhibitors (KIs) and antibody/peptide drug conjugates (ADCs/PDCs) were the most affected drug classes. Ten of the 21 approvals with dose-related PMCs/PMRs fulfilled their dosing PMCs/PMRs, and 3 of the 10 (30%) had relevant label changes.

**Conclusion:**

Most dose-related PMRs/PMCs were issued for NMEs. Of these, KIs and ADCs/PDCs were highly represented, reflecting their novelty and greater uncertainty around their safety profile. PMC/PMR issuance broadly increased over time. With the implementation of the FDA’s Project Optimus in 2021, it remains to be seen whether fewer dose-related PMCs/PMRs emerge in future due to enhanced dose optimization in the premarketing setting.

**Supplementary Information:**

The online version contains supplementary material available at 10.1007/s43441-023-00606-1.

## Introduction

Cancer treatment has dramatically evolved and improved over the years, moving from cytotoxic chemotherapy to more targeted therapies such as monoclonal antibodies (mAbs) and kinase inhibitors (KIs). One essential consideration for cancer treatment is dose selection, especially given the increasing diversity of therapies available for patients. Cytotoxic drugs generally follow a traditional dose selection process, since the exposure–response relationship is often linear (that is, higher doses of drug result in greater antitumor activity). Small cohorts of patients are exposed to increasing doses of drug until predefined severe or life-threatening toxicities (termed dose limiting toxicity or DLT) are observed. When a dose reaches DLT and stopping criteria are met, the previous dose is considered the maximum tolerated dose (MTD) [1–3]. This dose selection process enables physicians to maximize the administration of cytotoxic drug to patients with limited options.

However, newer targeted therapies can have a non-linear exposure–response relationship, demonstrating an earlier plateau in efficacy while toxicities may continue to increase [4, 5]. As a result, increasing doses beyond the optimal biologic dose may not enhance antitumor activity and only increase toxicity. In addition, novel therapies have extended survival in many patients and therefore can be administered for long durations. This increases the importance of assessing long-term tolerability, since intolerable toxicities may lead to poor adherence or premature discontinuation. New approaches are needed to optimize the doses of these novel anticancer agents [5, 6].

With this shift in the cancer treatment paradigm, the United States Food and Drug Administration (FDA) has guided study sponsors to evolve their approach to determine dose selection and dosing schedules. In 2021, the FDA’s Oncology Center of Excellence (OCE) initiated Project Optimus to ensure that doses of cancer drugs are optimized to maximize efficacy, safety, and tolerability. In order to develop new strategies for dose-finding and optimization, sponsors should leverage nonclinical and clinical data in dose selection based on parallel dose trials that extend for multiple dosing cycles, pharmacokinetic (PK) drug exposure, in vitro/in vivo receptor occupancy or target engagement, and utilization of modeling/simulation to predict outcomes by dose level [1, 2, 5, 7].

Given the renewed guidance to optimize dose selection in the premarketing setting, we sought to understand the impact of the FDA’s historical issuance of postmarket evaluations through Postmarketing Commitments/Postmarketing Requirements (PMCs/PMRs) to evaluate the optimal dose changes over time. We sought to quantify the outcome of these PMC/PMR studies and determine if they led to changes in dose strength, dose schedule, or safety language in the US prescribing information (USPI).

## Materials and Methods

All FDA oncology drug approvals from January 1, 2010, to December 31, 2022, were aggregated using the appropriate published literature [1] in addition to publicly sourced information from the FDA’s Oncology/Hematologic Malignancies Approval website [8]. PMCs/PMRs and the targeted dates of completion were collected from approval letters on the Drugs@FDA website [9]. Older data (from 2010 to 2015) were bolstered with published literature [1] and information presented during a 2022 FDA webinar on dose optimization [5].

Approvals of interest were identified by first searching for keywords—“dose,” “dosage,” “dosing,” “dose frequency,” and “dose schedule”—within the PMR and PMC sections of the approval letter. We only considered PMCs or PMRs for dose-related studies related to efficacy or safety signals and excluded dose-related studies to evaluate more routine populations (e.g., organized by race or hepatic/renal insufficiency) or drug–drug interactions (DDI).

For each therapy, we used Vivpro^AI^ (Vivpro, Lansdale, PA, USA) and the Drugs@FDA website to compare the label preceding the PMC/PMR targeted completion date to the label posted most recently after the completion date. For some applications, we could not find an exact PMC/PMR fulfillment label and/or date on the website since not all PMC/PMR studies are published. The most proximal product labels before and after the PMC/PMR fulfillment date were reviewed manually to identify changes or differences in language related to dose strength, dose schedules, adverse events (AEs), safety, and/or any efficacy. In cases where PMCs/PMRs were expected to read out but for which data from FDA's website were lacking, DailyMed was checked to identify whether any PMC/PMR fulfillments may have been reflected into the industry label, but did not yet make it onto the FDA website.

Label changes were grouped into 3 categories:

- Relevant Label Change (RLC): Label changes in dose strength, dose scheduling, dose modification, AE, safety, and/or efficacy verbiage
- No Relevant Label Change (NRLC): No label changes in dosing, dose scheduling, dose modification, AE, safety, and/or efficacy verbiage
- Not Applicable (NA): No label was found around the time of the PMC/PMR fulfillment date, the product or indication was withdrawn, and/or the study had not yet been completed

## Results

Dose-related PMCs/PMRs were analyzed in two time periods:

- 2010 – 2015: Limited data were available on dose-related PMCs/PMRs and their fulfillments for supplemental applications, largely due to the older age of the publicly available information. Therefore, data within this period were analyzed for new molecular entity (NME) approvals only.
- 2016 – 2022: These data were more comprehensive, allowing for a more in-depth analysis beyond the NME category alone. This period also included many novel therapies recently approved by the FDA, with some postmarketing studies still pending completion.

### 2010–2015

From 2010 to 2015, 103 applications were approved by the FDA oncology divisions, 42 of which were considered NMEs (Table 1). Of the 42 NME approvals, 33.3% (*n* = 14) had a dose-related PMC/PMR (11 PMR, 2 PMC, and 1 both PMC and PMR), and of these, 10 (71.4%) were proposed using the MTD as their initial labeled dose. Of the 14 NMEs with dose-related PMCs/PMRs, 6 (42.9%) resulted in a relevant label change, 6 (42.9%) did not, and 2 (14.3%) were considered NA (the product/indication the PMC/PMR was based on was withdrawn due to inability to complete the required post-approval studies) (Table 1).

**Table 1:** Number of FDA Oncology Approvals from 2010 to 2015

	2010	2011	2012	2013	2014	2015	Total
Total Approved Applications	9	12	20	17	18	26	103
First Approvals (NMEs)	2	7	11	8	9	5	42
NMEs with Dose-Related PMCs/PMRs (%)	1 (50%)	2 (28.6%)	4 (36.4%)	2 (25%)	2 (22.2%)	3 (60%)	14 (33.3%)

### 2016–2022

From 2016 to 2022, 314 applications were approved by the FDA oncology divisions, 94 of which were considered NMEs. Of the 94 NME approvals, 16% (*n* = 15) had a dose-related PMC/PMR. Of the 220 supplemental approvals, only 2.7% (*n* = 6) had a dose-related PMC/PMR (Table 2). Overall, there was a consistently increasing trend of the percentage of applications with dose-related PMCs/PMRs (Table 2).

**Table 2:** Overview of FDA-Approved Oncology Applications from 2016 to 2022

	2016	2017	2018	2019	2020	2021	2022	Total
Total Approved Applications	21	50	53	38	57	55	40	314
All Approvals with Dose-Related PMCs/PMRs issued	1	4	3	2	3	4	4	21
First Approvals (NMEs)	4	14	18	11	19	16	12	94
NMEs with Dose-Related PMCs/PMRs	0	3	1	2	3	2	4	15
Accelerated Approvals with Dose-Related PMCs/PMRs	0	1	1	2	2	2	3	11
Approved Applications with Dose-Related PMC/PMRs (%)	4.8%	8.0%	5.7%	5.3%	5.3%	7.3%	10.0%	6.7%

**Fig. 1:**
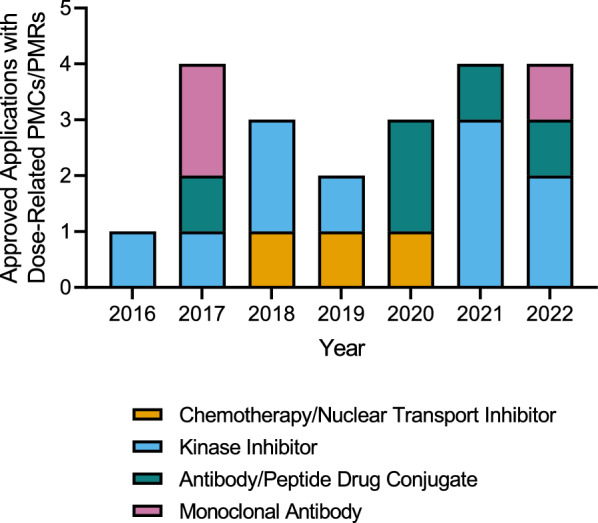
FDA-Approved Applications with Dose-Related PMCs/PMRs by Drug Class from 2016 to 2022

The approvals were categorized into 4 drug classes: monoclonal antibodies (mAbs), antibody/peptide drug conjugates (ADCs/PDCs), kinase inhibitors (KIs), and chemotherapy/nuclear transport inhibitors. Among these classes from 2016 to 2022, KIs comprised almost half (47.6%) of the applications that received a dose-related PMC/PMR upon approval, even though they were only 29.3% (*n* = 92) of the indications approved during this period. ADCs/PDCs were also notable since they comprised 23.8% (*n* = 5) of dose-related PMCs/PMRs, despite only being 8.3% (*n* = 26) of the total approvals from 2016 to 2022 (Fig. 1).

Since the target fulfillment date of PMC/PMR studies is typically years after approval, we only analyzed labeling changes from 2016 to 2019 (Table 3). Due to the recency of the data, most postmarketing studies in 2020 or later with dose-related PMCs/PMRs have not been completed or have yet to be submitted to the FDA based on the future PMC/PMR due dates listed in their approval letters.

**Table 3:** PMCs/PMRs Resulting in Label Changes from 2016 to 2019

	2016	2017	2018	2019
Applications with Dosing PMCs/PMRs	1	4	3	2
Label Change	0	2	1	0
Conversion Rate	0	50%	33.3%	0%

Between 2016 and 2019, there were 10 approved applications with dose-related PMCs/PMRs (Fig. 1, Table 3), 3 of which resulted in relevant labeling changes. No relevant label change was observed in 6 approved drug applications and 1 application is considered NA (due to a future, targeted PMC/PMR completion date). Of the applicable approvals, 3 of the 9 (33.3%) approvals with dose-related PMCs/PMRs resulted in labeling changes regarding dosing, dose schedules, qualifying AEs and safety, and/or updates to efficacy results (Fig. 1, Tables 2, 3).

## Discussion

Over the years, the FDA has recognized the need to incorporate clinically meaningful data into prescribing information for already-approved drugs. The FDA’s emphasis on safety and optimization is highlighted in the dose optimization cases of cabazitaxel and ceritinib (see Supplementary Information). Cabazitaxel was first approved on June 17, 2010. The FDA issued 2 PMRs for additional data that compared the approved dose with a lower dose to reduce hematological toxic effects and infections. On September 14, 2017, the FDA issued a supplemental approval based on the fulfillment of the PMRs, which lowered the recommended dose of cabazitaxel from 25 mg/m^2^ to 20 mg/m^2^. Similarly, for ceritinib, a KI approved on April 29, 2014, the FDA required a study comparing different doses in fasted versus fed states. On December 21, 2017, study results resulted in a label change from 750 mg with no food within 2 h and ceritinib discontinuation for patients unable to tolerate 300 mg with food, to a new label with a reduced dose of 450 mg with food and ceritinib discontinuation for patients unable to tolerate 150 mg with food.

With 16%, or 1 in 6, of NMEs approved from 2016 to 2022 having a dose-related PMC/PMR, the FDA has balanced the need to expeditiously review and approve novel therapies for patients with cancer with the uncertainty around optimal dose at the time of approval. There tends to be greater scrutiny in the postmarketing setting for novel therapies as they generally have less certainty around their safety profile and less long-term follow-up.

It is not surprising that a greater proportion (15 of 21; 71.4%) of dosing-related PMCs/PMRs from 2016 to 2022 were issued to KIs and ADCs/PDCs (Fig. 1, Table 2). KIs are well known for having off-target effects, particularly non-specific KIs that inhibit multiple kinase pathways simultaneously [10, 11]. These off-target effects may not only cause acute toxicities but also chronic AEs that may adversely affect the patient’s quality of life and result in the patient’s discontinuation of the therapy over time [10, 11]. Many ADCs/PDCs also have tolerability issues due to their narrow therapeutic index. These toxic effects often lead to skipped doses, dose reductions, or discontinuations due to the limited number of dosing cycles patients can tolerate [12, 13]. The FDA has made it clear that it is no longer to only assess DLTs when considering safety concerns, but sponsors must also assess low-grade, chronic toxic effects that may decrease patient adherence or drug tolerability [1, 2, 4, 5, 7].

**Fig. 2:**
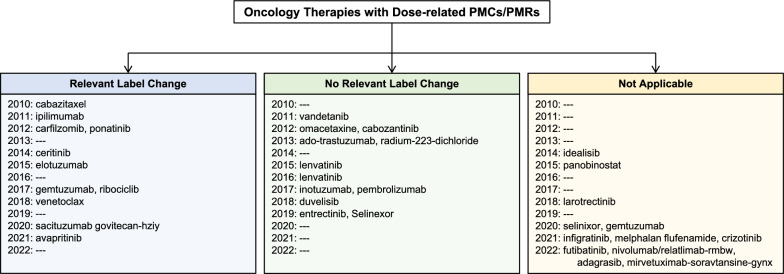
Label changes associated with dose-optimized PMCs/PMRs for oncology therapies from 2010 to 2022. Relevant label changes include changes to dose strength, frequency, modifications, safety, and/or efficacy. Not applicable includes no label found around the PMC/PMR fulfillment date, product withdrawal, or pending study completion

There has been a generally increasing frequency of PMCs/PMRs related to dose optimization over the years (Fig. 2, Table 4), from 5.3% in 2019 to 10% in 2022 (Table 2). Between 2016 and 2019, 3 out of the 8 (37.5%) dose-related PMCs/PMRs that have been fulfilled with relevant, supplemental published labels have resulted in labeling changes to their USPI. The noticeable increase of approvals with PMCs/PMRs in 2021 and 2022 may be attributed to KIs and ADCs, as 50% of KIs and 40% of ADCs from 2016 to 2022 received a dose-related PMC/PMR in 2021 and 2022 alone (Fig. 1). Moreover, Project Optimus was introduced in 2021, suggesting a stronger actionable commitment toward dosing optimization in the premarketing setting by the FDA, which may have also contributed to the recent increase in PMCs/PMRs.

**Table 4:** Outcomes of PMCs/PMRs for Oncology Therapy Indications from 2010 to 2022

Year	Drug	PMC/PMR Outcome
2010	Cabazitaxel	RLC
2011	Ipilimumab Vandetanib	RLC NRLC
2012	Carfilzomib Omacetaxine Cabozantinib Ponatinib	RLC NRLC NRLC RLC
2013	Ado-trastuzumab Radium-223-dichloride	NRLC NRLC
2014	Idelalisib Ceritinib	NA RLC
2015	Elotuzumab Lenvatinib Panobinostat	RLC NRLC NA
2016	Lenvatinib	NRLC
2017	Gemtuzumab Inotuzumab Pembrolizumab Ribociclib	RLC NRLC NRLC RLC
2018	Larotrectinib Duvelisib Venetoclax	NA NRLC RLC
2019	Entrectinib Selinexor	NRLC NRLC
2020	Selinexor Gemtuzumab Sacituzumab govitecan-hziy	NA NA RLC
2021	infigratinib Melphalan flufenamide Crizotinib Avapritinib	NA NA NA RLC
2022	Futibatinib Nivolumab/relatlimab-rmbw Adagrasib Mirvetuximab-soravtansine-gynx	NA NA NA NA

Relevant label changes include changes to dose strength, frequency, modifications, safety, and/or efficacy. Not applicable includes no label found around the PMC/PMR fulfillment date, product withdrawal, or pending study completion

*RLC* relevant label changes; *NRLC* no relevant label changes; *NA* not applicable

In January 2023, the FDA posted a draft dose optimization guidance that emphasized the need to collect the appropriate data regarding the dose–exposure response and other pertinent pharmacokinetic/pharmacodynamic (PK/PD) information to comprehensively assess the benefits versus risks for efficacy and safety/tolerability when developing new oncology drug and biological products [7]. Since novel oncologic therapies are changing the treatment landscape, the FDA expects sponsors to reassess the traditional approach toward dose-finding studies. Alternatives include more comprehensive dose-finding studies through multiple cohorts or expansions, using simulations and models, and a deeper understanding of the patient’s PK/PD to mitigate the risk of unnecessary toxic effects over multiple dosing cycles [2, 5, 7, 14].

One of the primary concerns from outside stakeholders with the FDA’s dose optimization guidance from Project Optimus is that enrolling additional patients to multiple dose levels without first demonstrating the therapy’s clinical activity will subject too many patients to ineffective agents while exposing them to unnecessary toxicities [15]. In addition, industry stakeholders have submitted comments to the FDA’s guidance highlighting the potential risk of exposing patients with life-threatening diseases to a subtherapeutic dosage, indicating a need for flexibility when recommending randomized studies of multiple dosages [15]. This debate indicates that there are some challenges ahead regarding managing speed, uncertainty, and patient risk–benefit profile to appropriately optimize a drug’s dose. Nonetheless, it is the new guidance around Project Optimus that will lead to more rigor and potentially longer timelines and larger dose-finding programs in the premarketing setting.

There were some limitations to this study. We could not confirm some of the PMC/PMR fulfillment dates due to potentially incomplete publicly sourced data, and therefore, the results might not comprehensively represent the landscape of dose-related PMCs/PMRs over the years. In addition, the exclusion of PMCs in special populations (e.g., by ethnicity, hepatic/renal insufficiency, or drug–drug interaction) might underrepresent the number of dose-related PMCs/PMRs for a particular drug. Because these populations may require modified doses or may be uniquely susceptible to dose adjustments, their inclusion in this analysis may artificially inflate the dose-related labeling changes associated with the primary indication population.

Overall, the effectiveness and flexibility of the FDA’s proposed dose selection strategies remain to be seen. FDA’s recommended dose optimization strategies have shown the potential to further evolve the drug development paradigm with the risk of extended premarketing drug development timelines, especially for therapies with newer mechanisms of action. Future studies will need to assess whether the increased timelines and rigor in the premarketing setting will reduce the number of postmarketing dose-related studies leading to relevant labeling changes.

## Conclusion

These results demonstrate that more PMCs/PMRs were issued over time and most dose-related PMRs/PMCs were for NMEs with new mechanisms of action, possibly reflecting their less-established safety profiles, which may impact their long-term tolerability. With the initiation of Project Optimus and more rigorous dose optimization in the premarketing setting, subsequent comprehensive analyses are needed to determine whether time to market will be impacted for NMEs, whether dosing PMR/PMCs will decrease over time, and whether postmarketing labeling modifications will be reduced over time.

### Supplementary Information

Below is the link to the electronic supplementary material.

Supplementary file1 (DOCX 617 KB)

## Data Availability

Merck Sharp & Dohme LLC, a subsidiary of Merck & Co., Inc., Rahway, NJ, USA (MSD) is committed to providing qualified scientific researchers access to anonymized data and clinical study reports from the company’s clinical trials for the purpose of conducting legitimate scientific research. MSD is also obligated to protect the rights and privacy of trial patients and, as such, has a procedure in place for evaluating and fulfilling requests for sharing company clinical trial data with qualified external scientific researchers. The MSD data sharing website (available at: http://engagezone.msd.com/ds_documentation.php) outlines the process and requirements for submitting a data request. Applications will be promptly assessed for completeness and policy compliance. Feasible requests will be reviewed by a committee of MSD subject matter experts to assess the scientific validity of the request and the qualifications of the requestors. In line with data privacy legislation, submitters of approved requests must enter into a standard data-sharing agreement with MSD before data access is granted. Data will be made available for request after product approval in the US and EU or after product development is discontinued. There are circumstances that may prevent MSD from sharing requested data, including country or region-specific regulations. If the request is declined, it will be communicated to the investigator. Access to genetic or exploratory biomarker data requires a detailed, hypothesis-driven statistical analysis plan that is collaboratively developed by the requestor and MSD subject matter experts; after approval of the statistical analysis plan and execution of a datasharing agreement, MSD will either perform the proposed analyses and share the results with the requestor or will construct biomarker covariates and add them to a file with clinical data that is uploaded to an analysis portal so that the requestor can perform the proposed analyses.
