# Supplementary material for: US FDA’s Dose Optimization Postmarketing Requirements and Commitments of Oncology Approvals and the Impact on Product Labels from 2010 to 2022: An Emerging Landscape from Traditional to Novel Therapies
Source: Ther Innov Regul Sci. 2024 Jan 5;58(2):380–6. doi: 10.1007/s43441-023-00606-1 (PMC10850176; doi:10.1007/s43441-023-00606-1)
Supplement: Supplementary file 1 — Supplementary file1 (DOCX 617 KB) [file 43441_2023_606_MOESM1_ESM.docx]

**Supplementary Information**

U.S. FDA’s Dose Optimization Postmarketing Requirements and Commitments of Oncology Approvals and the Impact on Product Labels from 2010 to 2022: An Emerging Landscape from Traditional to Novel Therapies

Joseph M. Gendy PharmD, Naomi Nomura MS, Jeffrey N. Stuart PhD, Gideon Blumenthal MD

Case #1: Cabazitaxel

Sanofi-Aventis received its first approval on June 17^th^, 2010 for the use of cabazitaxel Injection in combination with prednisone for the treatment of patients with hormone-refractory metastatic prostate cancer previously treated with docetaxel-containing treatment regimen ([Drugs@FDA Cabazitaxel Approval Letter With Dose-Related PMRs](https://www.accessdata.fda.gov/drugsatfda_docs/appletter/2010/201023s000ltr.pdf)).

- - PMR 1649-3: Conduct a Phase 3 randomized controlled trial in patients with hormone-refractory metastatic prostate cancer comparing 75 mg/m^2^ docetaxel with prednisone with cabazitaxel 25 mg/m^2^ with prednisone and cabazitaxel 20 mg/m^2^ with prednisone as first-line therapy. The primary endpoint should be overall survival to evaluate the incidence of drug-related death as well as efficacy. The trial should be powered to detect a 25% difference in overall survival. The trial will include interim analyses for evaluation of efficacy based on overall survival and safety of the 25 mg/m^2^ with prednisone arm versus the 20 mg/m^2^ with prednisone arm to potentially drop one of the cabazitaxel arms. Submit the protocol for agency review prior to commencing the trial.
  - PMR 1649-4: Conduct a Phase 3 randomized controlled trial in 1222 patients with hormone-refractory metastatic prostate cancer **previously treated** with docetaxel comparing cabazitaxel 20 mg/m^2^ with prednisone versus cabazitaxel 25 mg/m^2^ with prednisone and powered to preserve 50% of the treatment effect of cabazitaxel 25 mg/m^2^. The study will include interim analyses for evaluation of drug-related deaths and safety as well as overall survival of the cabazitaxel 25 mg/m^2^ with prednisone arm versus the cabazitaxel 20 mg/m^2^ with prednisone arm to potentially discontinue the trial.

On September 14, 2017, the FDA issued a supplemental approval based on their fulfillment of the PMRs sent in on November 14, 2016.

- New data resulted in the dose reduction of 25 mg/m^2^ to 20mg/m^2^ of carbazitaxel to reduce hematological toxic effects and infections


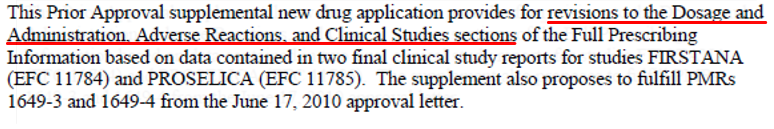


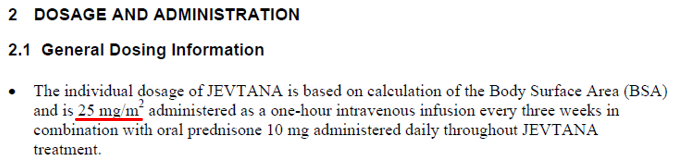


[Drugs@FDA Original Label - Cabazitaxel](https://www.accessdata.fda.gov/drugsatfda_docs/label/2010/201023lbl.pdf)


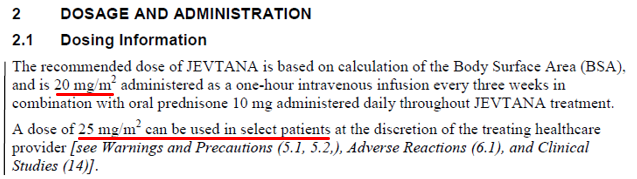


[Drugs@FDA New/Modified Label - Cabazitaxel](https://www.accessdata.fda.gov/drugsatfda_docs/label/2017/201023s019lbl.pdf)


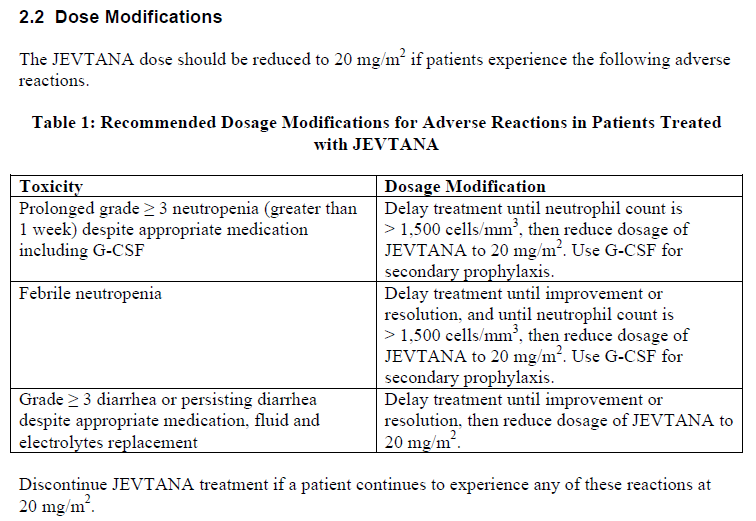


Drugs@FDA Original Label

(Same Link as Above)


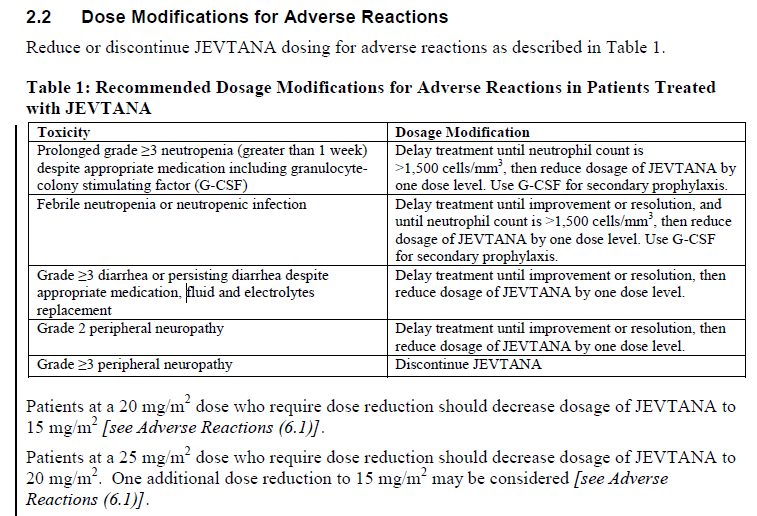


Drugs@FDA New/Modified Label

(Same Link as Above)

Case # 2: Ceritinib

Novartis received its first approval on April 29^th^, 2014 for the use of ceritinib as treatment of patients with anaplastic lymphoma kinase (ALK)-positive metastatic non-small cell lung cancer (NSCLC) who have progressed on or are intolerant to crizotinib ([Drugs@FDA Ceritinib Approval Letter with Dose-Related PMR](https://www.accessdata.fda.gov/drugsatfda_docs/appletter/2014/205755Orig1s000ltr.pdf)).

- PMR 2146-2: Conduct a clinical trial to evaluate the systemic exposure and safety of 450 mg Zykadia (ceritinib) taken with a meal and 600 mg Zykadia (ceritinib) taken with a light meal as compared with that of 750 mg Zykadia (ceritinib) taken in the fasted state in metastatic ALK-positive NSCLC patients.

On December 21, 2017, the FDA issued a supplemental approval based on their fulfillment of the PMR sent in on February 20, 2017.

- New data resulted in a label change from 750mg with no food within 2 hours and ceritinib discontinuation for patients unable to tolerate 300mg with food, to a new label with a reduced dose of 450mg with food and ceritinib discontinuation for patients unable to tolerate 150mg with food


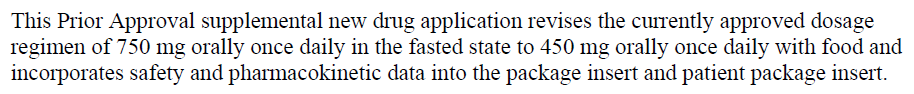


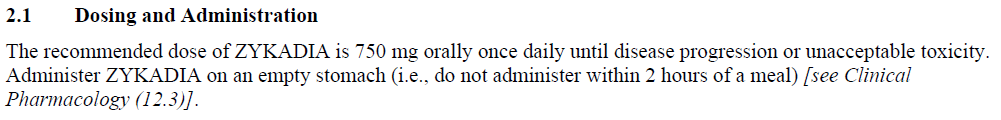


[Drugs@FDA Original Label - Ceritinib](https://www.accessdata.fda.gov/drugsatfda_docs/label/2014/205755s000lbl.pdf)


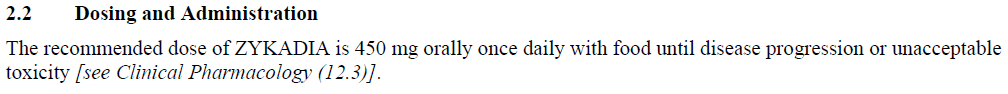


[Drugs@FDA New/Modified Label - Ceritinib](https://www.accessdata.fda.gov/drugsatfda_docs/label/2017/205755s010lbl.pdf)


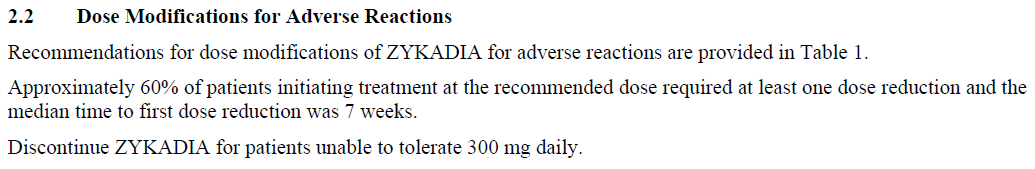


Drugs@ FDA Original Label

(Same Link as Above)


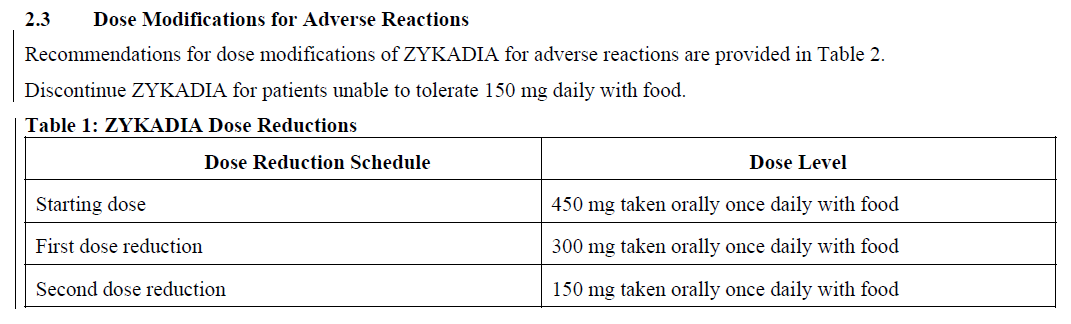


Drugs@FDA New/Modified Label

(Same Link as Above)
